# Non-Classical HLA Class 1b and Hepatocellular Carcinoma

**DOI:** 10.3390/biomedicines11061672

**Published:** 2023-06-09

**Authors:** Valli De Re, Maria Lina Tornesello, Vito Racanelli, Marcella Prete, Agostino Steffan

**Affiliations:** 1 Immunopathology and Cancer Biomarkers Unit, Centro di Riferimento Oncologico di Aviano (CRO), Istituti di Ricovero e Cura a Carattere Scientifico (IRCCS), 33081 Aviano, Italy; asteffan@cro.it; 2 Molecular Biology and Viral Oncology Unit, Istituto Nazionale Tumori IRCCS “Fondazione G. Pascale”, 80131 Naples, Italy; m.tornesello@istitutotumori.na.it; 3 Department of Interdisciplinary Medicine, School of Medicine, ‘Aldo Moro’ University of Bari, 70124 Bari, Italy; vito.racanelli1@uniba.it (V.R.); marcella.prete@uniba.it (M.P.)

**Keywords:** hepatocellular carcinoma, human leukocyte antigen, hepatitis virus, natural killer cells, Vδ2 T-cell, NKG2A receptor

## Abstract

A number of studies are underway to gain a better understanding of the role of immunity in the pathogenesis of hepatocellular carcinoma and to identify subgroups of individuals who may benefit the most from systemic therapy according to the etiology of their tumor. Human leukocyte antigens play a key role in antigen presentation to T cells. This is fundamental to the host’s defense against pathogens and tumor cells. In addition, HLA-specific interactions with innate lymphoid cell receptors, such those present on natural killer cells and innate lymphoid cell type 2, have been shown to be important activators of immune function in the context of several liver diseases. More recent studies have highlighted the key role of members of the non-classical HLA-Ib and the transcript adjacent to the HLA-F locus, FAT10, in hepatocarcinoma. The present review analyzes the major contribution of these molecules to hepatic viral infection and hepatocellular prognosis. Particular attention has been paid to the association of natural killer and Vδ2 T-cell activation, mediated by specific HLA class Ib molecules, with risk assessment and novel treatment strategies to improve immunotherapy in HCC.

## 1. Introduction

Hepatocellular carcinoma (HCC) is the most common type of liver cancer, accounting for 70% to 90% of all diagnoses, and expected to reach 22 million cases in the next two decades [1]. The main etiologic factors for HCC are hepatitis B (HBV) and C (HCV) viruses, steatohepatitis and metabolic syndromes, alcohol abuse and aflatoxin, while cirrhosis is considered as a major indicator for its surveillance [1].

HCC is still difficult to cure with a 5-year survival rate of less than 15%. In patients with advanced HCC, systemic therapies, including targeted therapy, immunotherapy, or a combination of both, are the mainstay of treatment [2]. However, immunotherapy used successfully in most refractory malignancies has not been as effective as expected in HCC, probably because of its particularly complex immune microenvironment. Indeed, antigen-rich blood from the gut passes through the liver, and this requires particularly efficient immune surveillance against pathogens and tolerance towards non-dangerous antigens [3,4]. The response rate for immunotherapy alone in a multi-center phase III study of nivolumab (an anti-programmed cell death protein 1, PD-1, drug) vs. the standard of care sorafenib (an anti-angiogenetic drug) confirmed efficacy only in 15% of patients [5]. The scientific community is now waiting for a better result from the combination of immune drugs (anti-PD-1 or anti-PD-L1 or anti-cytotoxic T lymphocyte-associated antigen 4 [CTLA4]) with multi-tyrosine kinases (anti-vascular endothelial growth factor receptor (VEGFR1-3), fibroblast growth factor (FGF) receptors (FGFR1-4), platelet-derived growth factor (PDGF) receptor α, KIT and RET. In the meantime, research on immune markers is ongoing to better understand the role of immunity in the pathogenesis of HCC, and to recognize which subgroups of individuals will most benefit from a systemic combined therapy according to liver cancer etiology [6].

To date, it is accepted that during HCC progression, the proportion of tumor-associated macrophages (TAM) displaying an M2 phenotype and expressing the immunosuppressive cytokines interleukin (IL)-10 and transforming growth factor-β (TGF-β) is increased, while cytotoxic natural killer (NK) and CD8 T cells are both decreased in number and inhibited through activation of immune checkpoints signaling (i.e., PD-1, CTLA4, TIGIT and LAG3) [7,8,9,10,11].

## 2. Classical HLA Class I Molecules (HLA-Ia)

The major histocompatibility complex (MHC), also known as the human leukocyte antigens (HLA), plays a key role in antigen presentation to T cells, leading to the basic formation of host defense mechanisms against pathogens, tumor cells and transplantation outcomes. HLA complex consists of more than 200 genes on chromosome 6, and can be categorized into three groups: class I, class II and class III [12].

Immunogenic peptides binding to HLA class I (e.g., HLA-A, B, C) are presented by nucleated cells and are recognized by cytotoxic CD8+ T cells; those binding to HLA class II (e.g., HLA-DR, DP, DQ) on the surface of the antigen-presenting cells [e.g., dendritic cells, macrophages, or B cells] and tumor cells display antigen peptide to CD4+ T cells, thus providing help to the effector cells such as innate immune cells, B cells and CD8 T cells [12]. Specific HLA alleles are the main risk of acute liver failure caused by certain drugs, by drug-metabolites and drug-modified peptides that can be recognized as foreign moieties trough the presentation of HLA-antigen complex. Examples are nevirapine in patients carrying the HLA-DRB1*0101 allele, flucloxacillin in HLA-B*5701 and minocycline in HLA-B*3502 carriers [13,14,15,16,17]. Furthermore, specific interactions of classical HLA with innate lymphoid cells (ILC) receptors, such as those expressed by natural killer cell (NK) and ILC2, have been shown to be important activators of liver-disease-associated immune function [18]. Among them, several studies have associated class I HLA (mainly HLA-A-Bw4 and HLA-C) with NK cell KIR receptors and the response to interferon treatment to induce HCV and HBV viral clearance, as well as the risk of developing HCC [19,20,21,22,23,24,25].

## 3. Non-Classical HLA Class I Molecules (HLA-Ib)

However, most recent studies have highlighted the key role played by members of the non-classical HLA class Ib (HLA-E, HLA-G, HLA-F, MHC class I related chain A (MICA/B) and Hla-F locus adjacent transcript (FAT10)) in HCC (Table 1).

It has been shown that antigen-presenting cells, as a consequence of infection or in response to antigen stimulation, produce several cytokines including IL-12 and IL-23, which are crucial for the secretion of IFN-γ mainly by T-cells, including CD4+ helper type 1 (TH1) cells, CD8+ cytotoxic cells and NK cells [51]. In addition, IFN-γ further increased the IL-12 production and secretion, thus creating a positive feedback loop between IL-12 and IFN-γ signaling [52].

**Figure 1:**
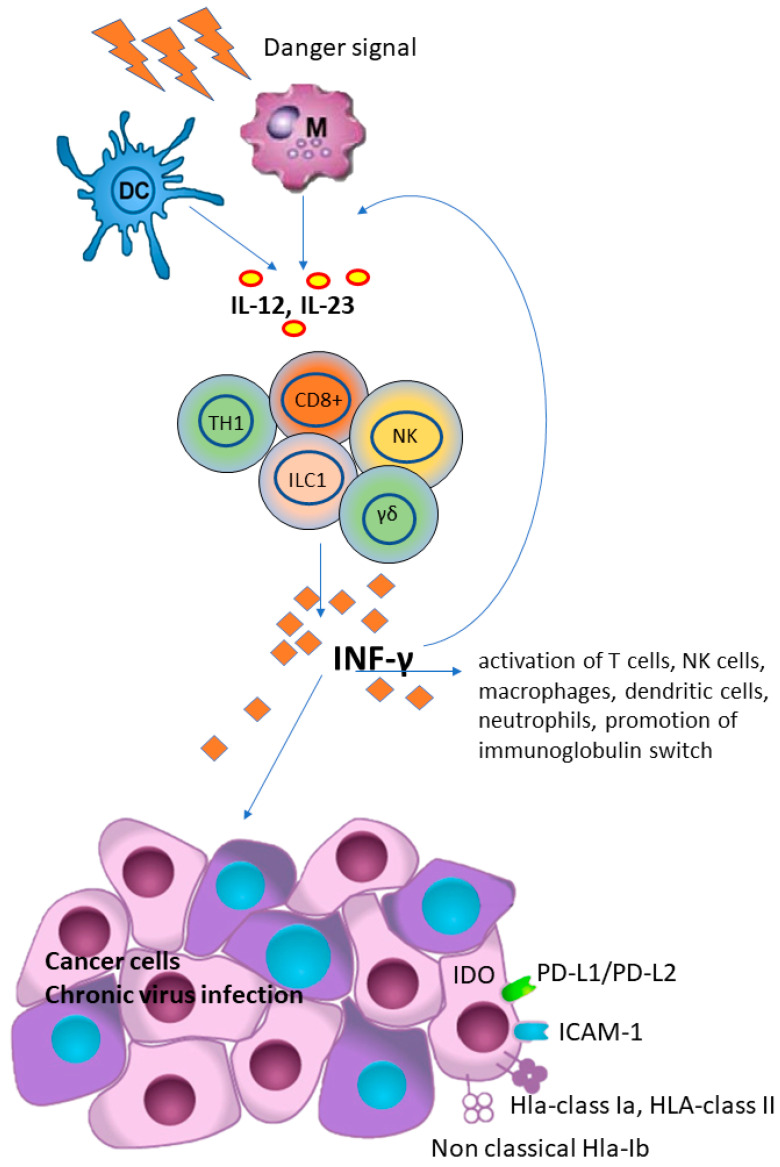
Interferon-gamma (IFN-γ) plays a key role in host defense and immune surveillance against microorganisms and tumor cells, primarily by acting as a potent activator of neutrophil and macrophage activities, promoting B-cell immunoglobulin isotype switching, promoting activation of CD8+ T-cells and differentiation of CD4+ T-cells towards a TH1 phenotype and, finally, up-regulating HLA class I expression and antigen presentation in tumor cells. However, IFN-γ has also been shown to have an immunosuppressive effect in tumors, as it promotes the PD-L1/PD-L2, indoleamine 2, 3-dioxygenase 1 (IDO 1), ICAM-1 and the expression of HLA and HLA-Ib (i.e., HLA-E, HLA-G and MICA) that suppress T-cells function. (DC), dendritic cell; (M), macrophage; (IL) interleukins 12 and 23; (CD8+), cytotoxic T cells; (NK), natural killer cells; (γδ), γδ T cells; (TH1), Th1 polarized CD4+ T helper cells; (ILC1), group 1 innate lymphoid cells; (HLA), human lymphoid antigen.

IFN-γ, a member of type II interferon, plays a key role in host defense and immune surveillance against microorganisms and tumor cells, primarily by acting as a potent activator of neutrophil and macrophage activities, promoting B-cell immunoglobulin isotype switching, promoting activation of CD8+ T-cells and differentiation of CD4+ T-cells towards a TH1 phenotype and, finally, up-regulates HLA class I expression and antigen presentation in tumor cells. However, IFN-γ has also recently been shown to have an immunosuppressive effect in tumors as it promotes the PD-L1/PD-L2, indoleamine 2, 3-dioxygenase 1 (IDO 1) [53,54], and the expression of HLA-Ia and HLA-Ib (i.e., HLA-E, HLA-G and MICA) that suppress T-cells function [55,56] (Figure 1). Therefore, similarly to other conditions present in different types of cancers [57,58], it has been proposed that cytokine secreted by NK cells, by modulating the immune response networks and counteracting viral infection, may be involved in liver fibrosis and poor HCC prognosis through the induction of a tolerant microenvironment [43,43,57,57,58,59,60].

Unlike classical HLA-Ia, the HLA-Ib molecules have a limited number of polymorphisms and low expression on the cell surface [12,61,62,63]. They bind receptors expressed on T, NK and NK-like T lymphocytes. In analogy to their function in trophoblasts, where they protect the fetus from maternal immune attack under special conditions, such as in the tumor microenvironment, their aberrant expression may promote the evasion of tumor cells from immune surveillance [63,64].

**Figure 2:**
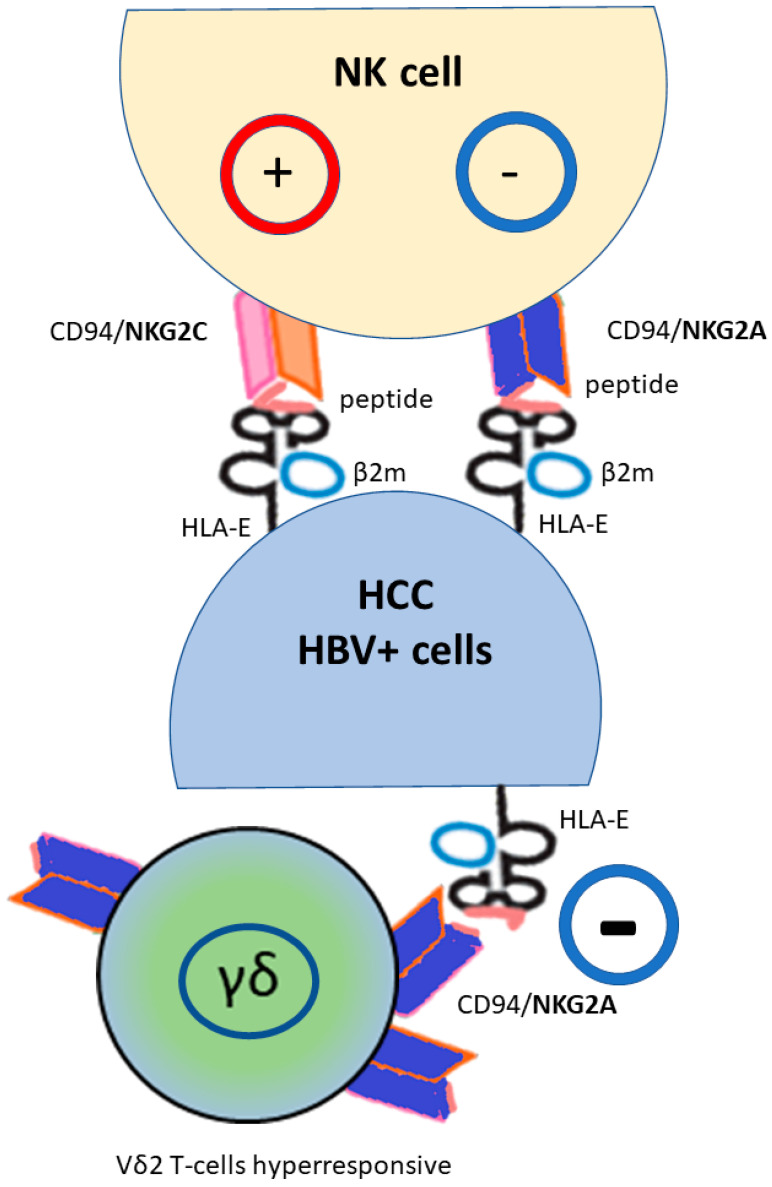
HLA-E molecule regulates natural killer (NK) and cytotoxic T-lymphocyte cells via the heterodimeric receptor CD94/NKG2, with a different effect on NK cells depending on the NKG2 type (activation NKG2C, inhibition NKG2A). Moreover, HLA-E*01:01 usually presents better virus-derived peptides than the HLA-E*01:03 that is more efficient at leading HLA class Ia leader peptide. NKG2A+ found a subset of Vδ2 T-cells educated to hyperresponsiveness; their function is inhibited by HLA-E expression. (HBV+) hepatitis B virus infected cells; (HCC) hepatocellular carcinoma.

HLA-E has only 2 alleles, namely HLA-E*01:01 and HLA-E*01:03, which differ in one amino acid at position 107, conferring significant differences in terms of charge, conformation, relative peptide binding affinity to selected peptides and, consequently, HLA-E expression on the cell surface [65,66,67]. Studies have also shown that the HLA-E molecule derived from the HLA-E*01:03 allele is more highly expressed on the cell surface and is more stable than the HLA-E*01:01 allele. This difference may result in a more effective inhibitory function of the HLA-E*01:03 allele in response to a wide range of ligands [68]. However, HLA-E can present peptides derived from the leader of some HLA-Ia molecules (as reported in the table [67]), but also other peptides such as the heat shock protein (hsp60) [69]. Furthermore, when HLA-E is bound to peptides derived from HLA leader molecules or some viral peptides, stimulation of the CD94/NKG2A receptor inhibits lysis and cells are protected from NK cell cytotoxicity, whereas presentation of the hsp60, and probably other peptides, results in loss of ligand recognition by the inhibitory NK receptor CD94/NKG2A and enables the lytic response of NK cells against stressed cells expressing the hsp60 antigen [70]. Thus, NK inhibition by the CD94/NKG2A/HLA-E system is peptide-specific. In addition, the HLA-E molecule regulates NK cells and cytotoxic T lymphocytes, with different effects on NK cells depending on NKG2 type expression (inhibition of NKG2A, activation of NKG2B, and NKG2C), activation receptors bind HLA-E with lower affinity than the inhibitory NKG2A receptor [68,69,70] (Figure 2). Interestingly, HLA-E, the only ligand for the inhibitory CD94/NKG2A receptor, whereas NKG2-activating receptors have multiple ligands, is frequently upregulated in many tumors [71,72,73,74,75,76]. HLA-E was found to be expressed on the surface of human hepatoma cells, resulting in negative regulation of NK cell activity through CD94/NKG2A interaction [77]. A higher level of HLA-E expression has been observed in HCC tissues compared to adjacent non-cancerous liver tissues and to normal liver [42,78,79]. Furthermore, NKG2A, but not NKG2C, was also upregulated on NK cells in tumor tissue from patients with HCC compared to patients without HCC, and higher NKG2A expression was associated with increased HLA-E expression [80]. These data suggest that HLA-E has a mainly inhibitory function in HCC, and that the NKG2A/HLA-E interaction may act as a resistance mechanism as a result of repeated activation of the immune system in the tumor microenvironment. NKG2A has been thus proposed as a marker for late tumor-infiltrating proliferating lymphocytes (TILs) [81,82] and increased expression was associated with exhausted NK functions [83] and reduced disease-free and survival rates [79]. Most importantly, the NKG2A/NKG2D ratio has been shown to be closely associated with HCC progression and immunosuppression [84], whereas switching to NKG2C on the NK cell surface indicates more mature NK cells [85,86]. A decreased NKG2D expression on circulating NK cells isolated form HCC patients compared to blood donors has also been observed [87]. Furthermore, the presence of inflammatory cytokines such as IL-10, IL15 and TGFβ, particularly abundant in the TME of HCC [88], has been shown to increase NKG2A expression on NK cells [88,89,90], and recently, a specific study of the gene signature once again suggested the CD94/NKG2-HLA-E system as the likely cause of the immune evasion of NK cells in HCC samples with higher levels of infiltrated NK cells characterized by the TGF-beta–Wnt interaction signature [91].

HLA-E is transcribed in most tissues but its expression on the cell surface is limited, and requires a peptide bound with a difference between the two HLA-E*01:01 and HLA-E*01:03 allele as consequence of their relative peptide affinity [66]. The peptide is mostly derived from the signal of HLA class I molecules, i.e., HLA-A, B, C and G, so the surface expression of HLA-E largely reflects the integrity of the antigen presentation pathway mediated by HLA class I molecules, or peptides derived from pathogens or cellular stress [92,93,94]. The peptides share a common motif: methionine at position 2, and leucine or isoleucine at position 9 [95], and these complexes can be detected by T cells via their antigen-specific T-cell receptor (TCR). It has been reported that the HLA-Ia leader sequence/HLA-E complex preferentially binds the inhibitory CD94/NKG2A receptor, while the HLA-G leader sequence/HLA-E complex binds the activating CD94/NKG2C receptor [96]. The viral peptide/HLA-E interaction with the CD94/NKG2 receptor was further complex. In fact, while viral infection caused CD94/NKG2A overexpression, the virus-induced reduction of HLA-Ia expression produced a minor HLA-Ia leader peptide/HLA-E complex with reduced interaction with the CD94/NKG2A receptor, resulting in virus-infected cells being able to be eliminated by CD8+ and NK cells. Moreover, in this context and as shown in susceptibility to COVID-19 infection, because HLA-E*01:01 allele is less efficient at leading HLA class Ia leader peptide than the HLA-E*01:03 allele, HLA-E*01:01 usually presents better virus-derived peptides than the HLA-E*01:03, thus affecting target cell recognition and cytolysis of virus-infected cells by CD8+ T-cells [97]. Consistent with these observations, in a study of HBV, an expanded population of NK-like CD8+ T-cells characterized by a HLA-E/CD94/NKG2C interaction was found in hepatic sinusoids, leading to activation of NK-like cells independent of TCR stimulation [98]. Because HLA-E expression increases following various cellular stresses, including cancer-related inflammation [28,99], it is probable that HLA-E/NKG2C binding is likely to strongly influence the immune response to eliminate infected cells. In addition, growing studies showed that a subset of unconventional gamma delta (γδ) T lymphocytes, harboring the Vγ9Vδ2 TCR (abbreviated as Vδ2pos) T cells, exert a potent antitumor activity in several cancers, and that the inhibitory NKG2A expression on these T cells may abolished the immune response, due to the HLA-E expression on the tumor cells in HCC [79,100] (Figure 2). Furthermore, patients with HBV infection had more exhausted NKG2A+ Vδ2+ T cells in the peripheral blood compared to uninfected patients [101]. The authors found that increased NKG2A expression is associated with more cytolytic cells, although direct stimulation by CD94/NKG2A is inhibitory. Thus, circulating NKG2A+ Vδ2+ T cells are functionally preserved in chronically HBV-infected patients, but have a suppressed function in acute infection. Interestingly, an in vitro blockade of the NKG2A receptor with a targeted antibody has been shown to restore the ability of Vδ2pos T cells even against HLA-Epos tumor cells. Thus, in general, high frequencies of NKG2A+ Vδ2 T-cells improve overall survival in HCC patients when they showed a decreased or a similar level of HLA-E compared to normal tissue. Based on these studies, and the evidence of educated and hyper-responsive NKG2A+ Vδ2 T-cells, HCC prognosis might be improved by the development of NKG2A-HLA-E checkpoint-based therapies.

HLA-G is more polymorphic than the other HLA class Ib antigens, with 117 alleles and 38 proteins [https://www.ebi.ac.uk/ipd/imgt/hla/about/statistics/, accessed on 5 June 2023]. It was first studied on the surface of extra villous cytotrophoblast cells in the placenta as a key factor that induces fetal immune tolerance during pregnancy [102]. HLA-G is an inhibitory molecule that binds immunoglobulin-like transcript 2 (ILT2), ILT4, killer inhibitory receptor 2DL4 (KIR2DL4), CD8 T-cells and CD160 receptor on NK and CD8+ T-cells [103,104,105,106,107]. LT2 binds to both classical HLA-Ia and non-classical HLA-Ib molecules. However, its binding affinity for HLA-G is three to four times higher than for the classical HLA molecule [107]. The interaction of HLA-G with ILT2 and ILT4 leads to inhibition of toxicity in monocytes/macrophages, maturation and activation of T cells in dendritic cells, phagocytosis in neutrophils, secretion of IFN-γ in NK, expression of chemokines and induction of apoptosis in T cells. Together, these effects serve to reduce the inflammatory response [39]. However, it is proposed that HLA-G expression on tumor B cells, as in Hodgkin lymphoma, may be potentially beneficial in controlling tumor cell proliferation, as in vitro HLA-G binding to ILT2 on B cell surface has been shown to cause inhibition of B cell proliferation, differentiation and antibody secretion [41]. In addition to its physiological role in maternal-fetal tolerance, HLA-G is commonly expressed by solid tumors and has been associated with poorer prognosis in several cancers [108,109], including HCC [110,111,112]. HLA-G expression has been reported in approximately 50% of primary HCC lesions, reaching 71% in advanced stage III disease. In addition, plasma-soluble HLA-G was found to be significantly higher in HCC patients than in normal controls [37]. In several studies, a neo-expression of HLA-G has also been observed in connection with a viral infection of the liver [35,113,114].

**Figure 3:**
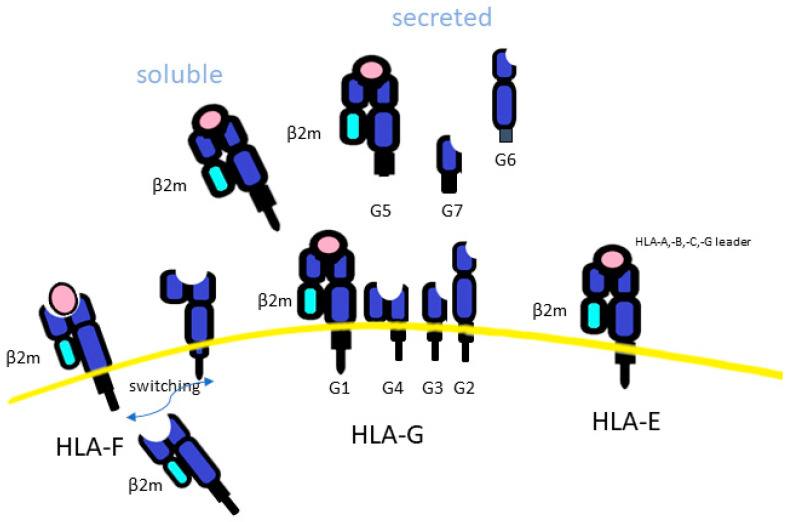
The difference among HLA class Ib. HLA-F, is an empty molecule, found intracellularly. HLA-F led to the classical trimeric closed HLA conformer form (beta2-microglobulin light chain (β2m), peptide and HLA-Ia) or an open conformation (because it is without a peptide). HLA-G may be secreted as a soluble form (sHLA-G), and this is associated with haplotypes mapping in the 3′UTR of the HLA-G genes.

The level of secretion of soluble HLA-G (sHLA-G) was associated with haplotypes mapping in the 3′UTR of the HLA-G gene [115] (Figure 3). Expression of sHLA-G has also been associated with HBV/HCV infection and poor prognosis, via increased viral load and fibrosis [35,78,112,116]. Indeed, it has been found that increased ILT2 expression engaging HLA-G contributes to the dysfunction of CD56dimCD16+NK cells [117] and that a tumor-expressing HLA-G promotes the accumulation and suppressive activities of immune cells such as Treg and mast cells, as well as converting NK cells, T-cells and macrophages toward a pro-tumor phenotype [118]. Moreover, sHLA-G, which is associated with the UTR1 HLA-G haplotype, was found tolerogenic, and thus reduces the inflammation also associated with a better clinical course in type 1 autoimmune hepatitis [119], as well as in liver transplantation through the immunosuppressive properties of HLA-G (reviewed in [35]). Of note, a recent study demonstrated NKG2A/CD94 to be an additional receptor for HLA-G that moreover was able to distinguish amino acid differences in the HLA-G heavy chain. In fact, G*01:04 allele was more protective against NK cell-mediated lysis than the other 2 allelic variants (G*01:04 and G*01.01) [120]. Overall, these results show sHLA-G and HLA-G protein as unfavorable markers in HCC prognosis, and they were potential targets for the development of new therapeutic strategies [33,34,36,37,111,121,122].

HLA-F, the smallest of the HLA class I molecules, had only 31 alleles, which can encode 6 distinct HLA-F proteins. HLA-F is an empty intracellular molecule, found mainly in the endoplasmic reticulum and Golgi apparatus (Figure 3). It acts as a ligand for transporters associated with antigen processing (TAP) and plays a role in the loading of endogenous peptides onto HLA class I molecules to CD8+ T-cell presentation and trans-interaction with NK receptors [123]. This led to the classical trimeric closed HLA conformer form (beta2-microglobulin light chain (β2m), peptide and HLA-Ia). However, HLA-F has also been reported to mediate HLA class I exogenous antigen cross-presentation [124]. In this case, HLA-Ia, which is in an open conformation (because it is without a peptide), can present its α1 domain free to interact with HLA-F [125,126]. Then, the HLA-I/HLA-F heterodimer is transported to the cell surface to present extracellular/foreign antigens after endocytosis to T-cells. This process has been observed to occur mostly during an inflammatory response, characterized by high levels of IFN-β, IFN-γ, and TNF-α, where overexpression of HLA-F and HLA-I in open conformation were shown in lymphocytes, monocytes and tumor cells [126,127,128,129,130,131,132,133]. In addition to HLA-peptide complex presentation to TCR, when classical HLA class I molecules were downregulated, thus reducing peptides presentation for immune recognition, HLA-F in open conformer may be exported on the cell surface and result in a marker of activated lymphocytes [134,135], conferring an inhibition of T and NK cell function [127]. It was then demonstrated that HLA-F in open conformation had a high affinity for the NK activator KIR3DS1 and a low affinity for the inhibitory receptors KIR3DL1 and KIR3DL2 [126,136,137], while HLA-F, expressed on the cell surface associated with β2 microglobulin, exhibited, as a consequence of peptide presentation, a reduced interaction between HLA-F and KIR3DS1. Moreover, NK cells from homozygous KIR3DS1 carriers showed a higher antiviral capacity compared to those carrying homologous inhibitory KIRDL3 homozygosity [138]. Overall, HLA-F in open conformer by activation of NK cell function has been associated with the resolution of some infections, including HCV and HIV infection, while hemoglobin peptides had been proposed to have a role in HIV progression, due to a reduction of HLA-F/KIR3DS1 interaction [138,139,140,141]. Previous studies, including one of ours, when the HLA-F function of NKs was still unknown, highlighted the potential role of balance between KIR3DL1 and KIR3DS1 on NK function and outcomes of HCV, HBV and HCC [19,24,25,142,143]. Although the clinical relevance of HLA-F expression in HCC patients is still unclear, HLA-F has attracted attention as an important immunosuppressive molecule, the expression of which was found to correlate with the degree of lymphatic or venous invasion in HCC [144].

More recently, a ubiquitin-like protein that targets proteins for degradation by being part of the ubiquitin-proteasome system (UPS), and thus affecting the peptide/HLA-I presentation, has been reported adjacent to the HLA-F gene region. This protein was called human leukocyte antigen F locus adjacent transcript 10 (FAT10), and has been associated with a susceptibility to inflammation-driven diseases, like nonalcoholic fatty liver disease (NAFLD), non-alcoholic steatohepatitis (NASH), and HCC [145]. Inflammatory stimuli, fibrogenesis and cytokines induced the expression of FAT10 [50,145,146,147]. FAT10 has been involved in the Mallory–Denk Bodies (MDBs), aggresomal molecules formed from undigested ubiquitinated short-lived proteins resulting from a reduced proteasome’s degradation rate, observed in hepatitis, alcoholic steatohepatitis, NAH and HCC [146,148,149,150,151]. Moreover, the upregulated expression of FAT10, related with IFNγ and TNF-α in hepatocytes, was found to accelerate the proliferation and progression of HCC-derived cells in vitro [152,153]. Conversely, FAT10 limits type I INF production in order to avoid exceeding inflammation and tissue destruction [154]. Based on all these results, FAT10 was proposed as a new biomarker in cancer, including the HCC [155,156].

## 4. NK and NK-like T-Cells

Cytotoxic CD8+ T-cells, natural killer (NK) cells and NK-like T-cells are effector lymphocytes involved in tumor immunosurveillance, and their abundance was lower in HCC tissues than in the adjacent normal tissues [80].

CD8+ T cell response is related to the tumor cell ability to recognize classic HLA-Ia antigen complex presentation to specific CD8+ T-cell receptors (TCR). Of note, tyrosine kinase inhibitors, used in HCC treatment, were also found to stimulate HLA-Ia expression in HCC, showing that downregulation of HLA is reversible [157,158]. However, the liver shows disrupted activation of most CD8+ T cells leading, in case of high antigen load, to antigen persistence accompanied by T-cell exhaustion and hepatitis development [159,160]. Tumor endothelial marker 1 (TEM1), epithelial cell adhesion molecule (EpCAM) melanoma-associated gene-A1 (MAGE-A1), New York esophageal squamous cell carcinoma 1 (NY-ESO-1), AFP and glypican-3 (GPC-3) have been identified as specifically associated with HCC and leading to a T-cell response in the blood in about 50% of patients [161]. These antigens are now used for the development of several CAR-T cells, an adoptive T-cell transfer therapeutic strategy, to avoid the involvement of TCR [e.g., Clinical Trials.gov Identifier: NCT03198546 and NCT02395250]. However, mainly due to the specific immunological tolerance of the liver as the organ that filters the blood effluent from the intestine [3], the efficacy of CAR-T cells in HCC showed a low efficacy if compared to hematologic and lymphatic cancer, and needs to be improved [162,163,164]. In addition, serious toxicities such as immune effector-cell-associated neurotoxicity syndrome (ICANS) or even life-threatening immune-related toxicities, such as cytokine release syndrome (CRS), are not uncommon with the use of the CAR-T approach, requiring multidisciplinary management with the need to develop algorithms that can identify patients at high risk of toxicity [165,166].

An alternative to CAR-T may be represented by innate immune cells like NK, gamma delta T-cells (γδT) and myeloid cells [167,168,169].

NK cells are highly enriched in the human liver, accounting for about 30% of the intrahepatic lymphocytes, and are highly associated with the outcomes of patients with HCC. The infiltration of NK cells into the liver increases further after surgery, due to the inflammation that occurs post-surgery, and this finding further supports importance of NK cells in the liver pathogenesis [170]. Unlike the effector function of CD8+ T cells, NK cells depend on a combination of activating and inhibitory receptors that bind primarily to HLA molecules in their peptide-binding region [Table 1]. The differences in receptors, both as genetic variation (allele) and in number, placed on individual NK cells lead to different repertories among individuals. Tolerance to one’s own molecules depends on the recognition of HLA antigens present on the target cells by the inhibitory receptors, especially KIR2DL, present on NK cells. Signals received through several inhibitory receptors during NK cell development promotes cell function, termed NK cell education [171]. Meanwhile, NK cells can sense the absence of HLA expression on cell surfaces, and some activating receptors such as killer-cell immunoglobulin-like receptors (KIR) and NKG2C receptors, which have variable expression over the time, can also detect HLA molecules as stress signals, causing the activation of NK effector functions. Moreover, it has been proved that activation of an NK receptor from differentially educated NK cells, for example by carrying more inhibitory receptors such as NKG2A or KIR3DL1 in addition to KIR2DL, showed enhanced NK cell activity [83]. Therefore, modulation of HLA expression and changes in the peptide-HLA immunocomplex repertoire may influence the functional activity of NK cells, resulting in a complex relationship between tumor microenvironment, difference in NK cell formation and expression of activating NK receptors [62]. In addition, the expression of gene encoding for immune checkpoint proteins, such as KLRD1/HLA-E, PD-L1/CD274/PD-CD1 and CTLA4/CD86, may in turn influence NK cell activation such as cytolysis, antibody-dependent cellular cytotoxicity (ADCC) and cytokine release, their survival, and the abundance and inhibition of NK cells in patients with HCC. In the end, as for the increase in activated CD8+ T cells, increased number of NK cells was found to prolong both relapse-free and overall survival of HCC patients [62].

The main strategies employed by viruses to evade NK cells are the reduced expression of ligands for activating receptors or the increased levels of inhibitory ligands. After viral infection, a variety of stress-induced molecules are expressed on the surface of infected cells, which can be recognized by activating receptors on NK cell receptors; the expression of HLA-C, a molecule able to interacts with the inhibitory KIR2DL receptor, is among the molecules most used by a virus to supply a reduction of NK functions. As different combinations of HLA ligands and KIR receptors in HCC have been shown to produce a different responses to treatment with anti-PD-1 immunotherapies in patients with virus-related HCC compared to non-alcoholic steatohepatitis (NASH) or controls, data had supported this concept [57].

More recently, a distinct population of NK-like hepatic CD8+ T-cells, characterized by a CD56 hi CD161−CD8+ phenotype and activated by a NKG2C/HLA-E complex independent from TCR, had been reported expanded in the liver sinusoids of the liver, and showed a strong immune response [98].

## 5. Non-Classical NKG2A HLA-E as a Novel Potential Checkpoint Targeted Therapy for HCC

Researchers have recently turned their attention to immunotherapy, as chemotherapy and radiotherapy have shown little survival benefit for HCC patients. Previous studies have confirmed that levels of tumor-infiltrating CD8+ T leukocytes are associated with better prognosis for both overall survival and disease-free survival in HCC patients, but most are inhibited by receptor-ligand interactions [172]. The most-studied targets of immune checkpoint inhibitors (ICIs) are the programmed cell death receptor (PD-1) on T cells and its ligands PD-L1 and PD-L2 on tumor cells. However, only 15–20% of patients have shown benefit from anti-PD-L1 monoclonal antibodies, which inhibit interactions with PD-1 and restore the role of T cells in the TME [173,174]. In addition, due to their toxicity, biologic drugs should be used with caution, especially in liver transplant patients [175].

Indeed, combination therapies, including PD-1/PD-L1 ICIs, have shown promise and several trials are ongoing in advanced or unresectable HCC. Among these, a combination of anti-PD1 with SP94-PB-sorafenib-Cy5.5 nanoparticles, a tyrosine kinase inhibitor (TKI), plus near-infrared therapy has shown suppression of distant metastases [176] and, in patients with early HCC recurrence after radical resection, TKI plus PD-1 therapy had shown survival benefit [177]. Data from IMbrave150, a phase III first-line trial in unresectable HCC, showed that after a median follow-up of 15.6 months, atezolizumab (anti-PD-L1) in combination with bevacizumab (anti-angiogenetic VEGF drug) reduced the risk of OS by 34%, with a median OS of 19.2 months, compared to 13.4 months for the TKI sorafenib. The next study, the HYMALAYA trial, approved in 2022, combined two different ICIs, the durvalumab [PD-1/CD80(B7.1), PD-L1 ICI] and tremelimumab (CTLA-4 ICI), in comparison to the sorafenib. The results of the study showed an OS of 16.4 months versus 13.8 months (*p* = 0.0035) after a median follow-up of 33.2 months, with a notable durable response. For advanced HCC, immunotherapy has now become the standard of care. A comprehensive characterization of the molecular patterns associated with response and resistance in patients with advanced HCC treated with anti-PD1 has recently been reported, and therefore has the potential to maximize the efficiency of anti-PD1 application [178]. However, phase III trials are needed to assess the ultimate value of this signature.

In addition to CD8+ T-cell inhibition, lower levels of NK cell infiltration have been found in HCC and more often in recurrent tumors in comparison to adjacent normal tissues [91]. Specific expression levels of NK cell immune checkpoint gene signatures highlighted a high correlation with the CD94/NKG2-HLA-E system as the probable cause of immune evasion in HCC with higher infiltrated NK cells, which are characterized by TGF-beta and Wnt interaction signature, in the HCC tissue. In addition, CD94 and KIR2DL4 (activation receptor for HLA-G) were highly expressed in patients responsive to targeted therapy with sorafenib, a kinase inhibitor.

Although liver transplantation and hepatectomy remain successful therapeutic strategies for patients with recurrent HCC, cytokine-induced killing (CIK) cell-based immunotherapy using ex vivo expanded T lymphocytes expressing the T-cell markers CD3+ or CD3−CD56+ have gained popularity as a promising new adjuvant therapy approach, targeting both exhausted T cells and NK cells to reduce HCC recurrence rates [179,180].

Several clinical trials have shown that CIK cell-based immunotherapy increases RFS in HCC patients who have undergone surgical resection (reviewed on [181]). The 18-month recurrence rate after adjuvant CIK therapy was 15.6%, compared to 40.0% in the control group receiving ablation therapy alone [182]. The rationale is that the irreversible cellular injury caused by ablation (RFA or cryoablation) allows an inflammatory TME with release of tumor neoantigens. However, the benefit of neoantigens in priming the immune response against tumor cells is hindered by the intrinsic inhibition of tumor cells. Therefore, the combination of ablation and CIK may synergize with the benefit of ablation.

Recently, the inhibitory NKG2A/HLA-E receptor has received attention as a new type of immune checkpoint blocker, and has also been proposed to disrupt the escape of circulating tumor cells from solid tumors [183]. In addition, it has been shown that HLA-G is also a ligand for the CD94/NKG2A receptor, but with a different affinity for the different HLA-G variants [120]. NKG2A blockade as a standalone therapy has appeared poorly effective in mouse tumor models; however, in the presence of activated T cells, for example, induced by PD-1/PD-L1 blockade, it exerts strongly enhanced efficacy. Clinical trials showed the safety of the humanized NKG2A-blocking antibody, monalizumab, and the first results of the phase II study had proved encouraging durable response rates in gynecologic cancers and head and neck carcinoma, including regressions of target lesions in recurrent disease. An increased effect for monalizumab has been shown by an enhanced ADCC function of NK cell and CTL cell effector, indicating that NKG2A-blocking directly impacts on NK cell effector functions [184,185].

Granulin-epithelin precursor (GEP), a hepatic oncofetal protein, was found to up-regulate NKG2A and HLA-E in the HCC microenvironment to enhance HCC cell invasion and resistance to human NK cell cytotoxicity, while downregulated MICA (MHC class I chain-related molecule A), the ligand for the stimulatory receptor NKG2D [186], a blockade of GEP by monoclonal antibody was demonstrated able to sensitized HCC cells to NK cytotoxicity. It was also shown that, as liver cancer progresses, the beneficial effect of stimulatory NKG2D immune responses can be also inhibited by increased expression of inhibitory receptors influenced by tumor-related metabolic disruption (tumor glycolysis and oxidative metabolism) and by tissue/cell mechanical properties, such as pathological conditions that drastically alter tissue stiffness, such as HCC [186,187,188].

In recent years, adoptive cell therapy has become one of the pillars in the treatment of cancer. One of the most important branches of this therapeutic approach, chimeric antigen receptor (CAR-T) therapy, has shown remarkable efficacy in the treatment of hematological cancers, solid tumor derived cell lines and in patients with glioblastoma but with an attention to severe toxicity [189,190].

Based on the observation that TGF-β increases the expression of the inhibitor NKG2A in HCC, a CAR NK cell engineered to convert the TGF-β-induced suppressive signal into an activating signal was developed [88,90]. Interestingly, these cells were better attracted to TGF-β-expressing tumor cells, inhibited the differentiation of human naïve CD4+ T cells into Treg cells, and could inhibit tumor growth in vivo in a xenograft model of HCC. Several clinical trials (NCT02906397, NCT02947165, NCT02423343, NCT02178358NKGD2) of monoclonal antibodies or CAR-T cells for TG-B blockade in HCC have been completed or are currently ongoing.

NKG2D-based CAR-T and CAR-NK cells have also been developed. In preclinical studies, they proved potent cytotoxicity against NKG2DL+ cell lines in vitro, therapeutic activity against NKG2DL+ cell xenografts in vivo and were shown to be safe. Two phase I trials were ongoing in patients with relapsed/refractory NKG2DL+ solid tumors, with a focus on NKG2DL+ HCC. [NCT05131763; NCT04550663].

The development of new therapeutic approaches for HCC through a targeted blockade of the NKG2A/HLA-E interaction of Vδ2pos T cells is of particular interest today. NKG2A has been shown to play a role in the development of NK cells. Indeed, NKG2A/HLA-E interaction makes NK cells educated and hyperreactive, increasing their effector functions in response to stimulatory activation. Conversely, NK cells that fail to interact because they lack NKG2A are poorly educated and unresponsive. However, educated NK cells are inhibited by the aberrant expression of HLA-E on HCC cells, resulting in an inhibitory function on Vδ2pos T cells, which are also unable to perform ADCC and escape immunological surveillance by the tumor. In preclinical studies, blocking the NKG2A receptor with a targeted antibody has been shown to restore the capacity of Vδ2pos T cells even against HLA-E+ tumor cells. It is thought that a sizable number of NKG2A+ Vδ2 T cells may improve overall survival in HCC when the level of HLA-E is reduced, or similar to, normal tissue. Based on these studies and the evidence that educated NKG2A+ Vδ2 T cells are more responsive, the prognosis of HCC may be improved by the development of therapies based on NKG2A-HLA-E immune checkpoint inhibition [191,192]. Most studies are ongoing, but currently only in preclinical models and in different cancer types, e.g., [193,194,195,196]. However, this new immune approach, NKG2A/HLA-E ICI, appears promising for patients with HCC, and the biological evidence obtained so far in vitro is encouraging.

## 6. Conclusions

This review reports on the increasing understanding of the key role of non-classical HLA-1b molecules in HCC pathogenesis and prognosis. Based on the current data, clinical applications of HLA-Ib in HCC risk assessment have been proposed.

Today’s findings identify NKG2A/HLA-E as an immune-checkpoint inhibitor and it seems the most promising target for new therapeutic approaches involving the HLA-Ib antigens to improve NK and, in particular, Vδ2 T cell function. Promising preclinical results have been obtained by combining NKG2A inhibitors with existing ICI therapies such as PD-1/PD-L1, multi-kinase inhibitors or anti-angiogenics. A NKG2D-based CAR-T cell therapy has been developed for the treatment of relapsed or refractory NKG2DL+ solid tumors [197] and a phase I clinical trial focus on HCC is ongoing [NCT04550663; NCT05131763].

The information on HLA-G and, in particular, HLA-F and FAT10, is too recent to be translated into therapeutic approaches at present. However, it would not be surprising if this were to happen in the near future, given the important information that has recently been reported on tumors and infections involving these molecules.

However, due to the limited number of studies available on the association between more recent HLA-Ib discoveries, compared to classical HLA class I and clinical outcome in patients with HCC, further research is needed to be more conclusive on these issues.

Taken together, the evidence described demonstrates that NK cells are dysfunctional in HCC, that their revitalization appears to be feasible, and these findings are driving the development of novel ICIs aimed at harnessing NK cell-mediated effector functions.

## Figures and Tables

**Table 1:** NK-like and NK cell receptors binding classical and non-classical HLA class I.

	Receptors	Ligands	Reference
Inhibitory receptors	KIR2DL1	HLA-C2	[26]
	KIR2DL2	HLA-C1	[27]
	KIR2DL3	HLA-C1	[21]
	KIR3DL1	HLA-Bw4	[19,28]
	KIR3DL2	HLA-A3, HLA-A11, HLA-B27	[29,30]
	KIR2DL4	HLA-G, soluble HLA-G	[31,32,33,34,35,36,37]
	CD94/NKG2A	HLA-E, HLA-G	[1,2,3,4,5,6]
	ILT2, ILT4, CD8	HLA-A, B, C, G, soluble HLA-G	[38,39,40,41]
Activating receptors	CD94/NKG2D	HLA-E, HLA-G, MIC-A/B	[2,3,12,33]
	CD94/NKG2C	HLA-E, HLA-G	[31,35,42,43]
	KIR2DS1	HLA-C2	[44]
	KIR2DS2	HLA-C1	[44]
	KIR2DS4full isotype	HLA-A11, HLA-C05/epitope specific	[45,46]
	KIR3DS1	HLA-F open form, HLA-F/HLA-1 heterodimer open form	[47]
	NKp30	HLA-B7	[48]
Proteasome	Target proteins for degradation mRNA protein	HLA FAT10 (HLA-F-adjacent transcript 10)	[49,50]

Activating receptors: recognize cell surface ligands expressing diverse types of stress, non-self, dangerous signals, while inhibitory receptors recognize self, non-dangerous signals. Killer-cell immunoglobulin-like receptor (KIR); MHC class I related chain (MIC-A/B); Ig-like transcript 2/4 (ILT).

## Data Availability

All data was in the text.
